# Cannabis and Polypharmacy Leading to Hospitalization for Acute Confusion and Inability to Ambulate

**DOI:** 10.7759/cureus.80481

**Published:** 2025-03-12

**Authors:** Lucas McKnight, Joshua J Widman

**Affiliations:** 1 Internal Medicine, The Ohio State University Wexner Medical Center and Nationwide Children's Hospital, Columbus, USA; 2 Hospital Medicine, The Ohio State University Wexner Medical Center, Columbus, USA

**Keywords:** acute encephalopathy, fall risk, gabapentin, inability to ambulate, marijuana use, polypharmacy, tetrahydrocannabinol (thc)

## Abstract

Inability to ambulate is a common issue for which an Emergency Department (ED) may request inpatient admission to the hospital. Frequent contributing factors include advanced age, spinal stenosis, osteoarthritis, deconditioning, and polypharmacy. We present an interesting case of a patient admitted due to inability to ambulate who had a significant acute decline in function coincident with the introduction of gabapentin in the setting of heavy tetrahydrocannabinol (THC) use.

## Introduction

Admission requests for inability to ambulate, falls, and/or confusion are extremely common in hospital medicine. Often, patients have comorbid conditions such as hypertension, diabetes mellitus, obesity, cancer, deconditioning, and myopathy [1]. Additionally, recent hospitalizations and/or comorbid degenerative musculoskeletal conditions such as spinal stenosis or osteoarthritis often contribute [2]. Polypharmacy is a known concern in the elderly population [3]. Frequently, a combination of factors contributes to presentation. Other less common but notable conditions that may lead to this presentation include spinal cord compression, stroke, primary neurologic disorders, and medication and substance abuse-related adverse effects [2,3]. As with any patient presentation, it is important to take a thorough history and perform a complete physical exam so as to avoid premature closure of the differential diagnosis and potentially miss an important etiology for the inability to ambulate.

## Case presentation

A 74-year-old male with a past medical history significant for psoriatic arthritis (on leflunomide), coronary artery disease status post percutaneous intervention in the early 2000s, and essential hypertension presented to the Emergency Department (ED) via ambulance. He had reportedly sustained a low-velocity mechanical fall and was subsequently unable to get off the ground independently. Initially, Emergency Medical Services (EMS) was called and assessed him in his apartment. He had normal vital signs and oxygen saturations on assessment. EMS recommended transport to the ED for evaluation, but he refused transport to the ED at that time. An hour later, a home health nurse (caregiver for his wife) arrived and convinced him to go to the ED for assessment.

History obtained in the ED revealed that the patient's legs felt acutely weak, and he had lowered himself to the ground. He denied any falls. From that position, he was unable to return to standing on account of generalized weakness. The patient had no loss of consciousness at any point during this episode and maintained recall of the entire episode. He denied any recent fevers, lumbar pain, sensation loss, or bowel or bladder incontinence or retention. His daughter was present in the ED and had observed that the patient seemed to be more confused and weak in the last few weeks. Of note, he had a similar admission about one week prior for generalized weakness and acute confusion, with symptoms resolved prior to discharge. It was postulated that the recent addition of gabapentin (started one to two weeks prior for neuropathic low back pain) was contributing, and this medication was decreased from 100 mg three times daily to 100 mg twice daily at the time of discharge.

Further history revealed that he smokes marijuana or consumes marijuana edibles for recreational purposes daily (or sometimes both). It was difficult to quantify tetrahydrocannabinol (THC) use, but both he and his daughter agreed it was "extensive". His daughter added that he drinks two double martinis (four total standard drinks) about three to four days of the week. His medication list included leflunomide 20 mg daily, gabapentin 100 mg twice daily, and nifedipine 30 mg daily.

On admission, he had normal vital signs and oxygen saturation. An extensive neurologic exam was performed. His cranial nerve functions were intact, and sensation was intact throughout. He had significant difficulty when instructed to move from supine on the bed to an upright seated position with legs hanging off the side of the bed but was ultimately able to accomplish this. From there, he was able to stand with significant instability. Rapid alternating movements were slow and unsteady. His motor strength was 5/5 in all major muscle groups without signs of focal deficits. He was appropriately oriented to place, time, self, and situation and was able to provide the history, but his daughter noted that he seemed slow and a little confused compared to his baseline. Physical examination was otherwise unremarkable.

**Figure 1:**
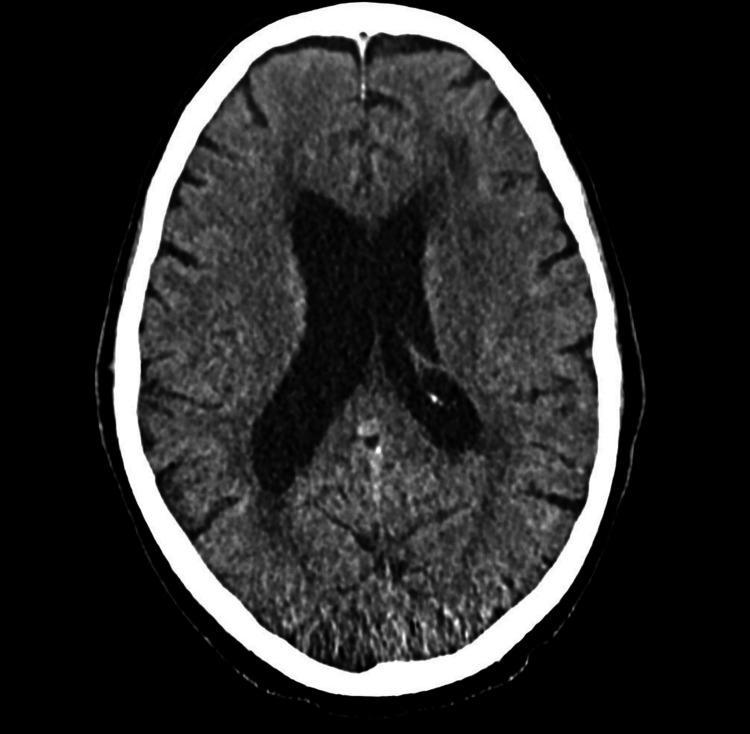
Head CT showing no acute intracranial findings.

During his most recent admission, a head CT was obtained and was negative for acute disease (Figure 1).

**Figure 2:**
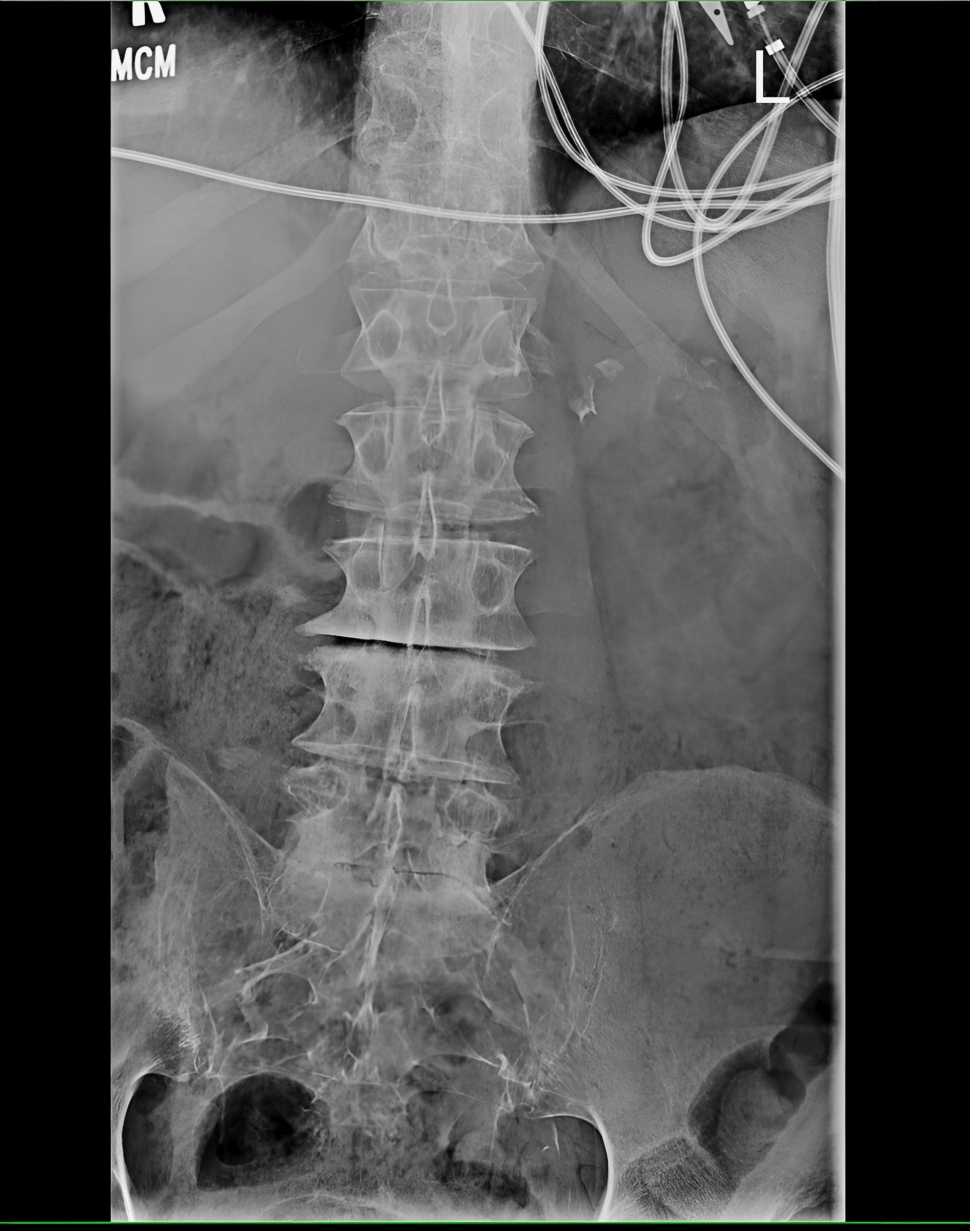
The lumbar spine X-ray demonstrates multilevel degenerative disc disease and facet arthrosis, most severe in the lower lumbar spine. No compression deformity or malalignment is observed.

Plain films of the lumbar spine were done on this admission, which showed lumbar spine degenerative disc disease but no compression deformities or likely etiology of his acute weakness (Figure 2).

His labs from the ED were largely unremarkable, including a bland urine analysis, normal chemistry panel, and complete blood counts without leukocytosis or clinically significant anemia. Further workup on admission included normal vitamin levels for thiamine, B6, B12, and folate. His thyroid studies were reassuring. The ethanol level was undetectable. His toxicology drug screen was positive for cannabinoids, with a quantitative THC metabolite level greater than 500 nanograms/milliliter (consistent with heavy use).

He was admitted to the observation unit. By the following morning, he and his daughter both felt his mental status was improved to baseline. He was seen by a neurologist, who noted that he was back to his prior baseline, able to ambulate with a walker and bear weight unassisted, and that his neurologic exam was non-localizing. His neuropathy and weakness were felt to be most likely due to neurological depression from the combination of marijuana use and gabapentin, given the fast return to baseline after stopping both drugs while in the hospital. Neurology recommended minimizing the use of central nervous system depressants, including marijuana, and to stop gabapentin altogether.

He was discharged with home healthcare services and counseled extensively to work with his primary care physician on stopping marijuana use. Gabapentin was stopped on discharge. Per chart review, he has had no further ED visits in the subsequent six months.

## Discussion

Cannabis is one of the most widely used substances in the world, with 2.5% of the world population reporting use in the last year [4,5]. In North America, use is even higher, with 45% of individuals in the US reporting at least one lifetime use and 18% reporting using at least once in 2019 [4,6]. With the trend in the US toward legalization, there is concern that increased access and lower cost may lead to increased health-related impacts [4,7]. After legalization in Colorado, there was a spike in emergency service utilization [4].

Gabapentin was first introduced as an anti-epileptic but has since been recommended for treatment of chronic neuropathic pain conditions [8]. The use of and prescriptions for gabapentin are also very common. An estimated 40,141,486 prescriptions were written in 2022, equating to almost 10,000,000 patients using the medication [9].

The combined usage of gabapentin and cannabis may increase side effects, including dizziness, drowsiness, confusion, and difficulty concentrating. Some patients may also experience impairment in thinking, judgment, and motor coordination [10].

Upon further review, our patient had a fairly classic presentation when combining cannabis and gabapentin. However, he sought medical care at least two times prior to a definitive recommendation to stop the combined use of both substances, indicating an opportunity for earlier recognition of the potential adverse effects of concurrent cannabis and gabapentin use. Even after extensive discussion and counseling with the patient, he was unable to commit to stop cannabis use or even decrease in usage. As such, given his presentation and the aforementioned risks associated with concurrent use, the decision was made to discontinue prescription gabapentin to mitigate the risk of recurrent adverse effects and potential readmissions. Additional efforts to reduce or discontinue cannabis use with help from his primary care physician were also advised. An interim review of his record shows no further ED visits or admissions in nearly six months.

Treating cannabis use disorder can be quite challenging, and abstinence rates are modest and tend to decline after the completion of treatment [11]. The treatment goal for cannabis use disorder may be either sustained abstinence or reduced use that mitigates cannabis-associated problems (a harm reduction strategy) [12,13]. It is our opinion that there needs to be further public education regarding the challenges associated with cannabis use disorder and addiction, and further research to improve treatment strategies given the potential for adverse effects with commonly used prescription medications as demonstrated by this case.

## Conclusions

This case highlights the complex interactions between illicit substances and prescription pharmacologic agents (in this case, cannabis and gabapentin) in an elderly patient. The patient’s presentation of acute weakness and confusion was ultimately attributed to the combined CNS depressive effects of these agents on his mentation and ability to ambulate. Despite multiple interventions and counseling, the patient was unable to commit to discontinuing or reducing cannabis use. Cannabis cessation remains a significant challenge for many patients. The management of cannabis use disorder in older adults presents unique challenges, such as the increased coincidence of prescription drug use, as this case demonstrates. This case underscores the importance of considering the interactions of prescription medications and substance use when evaluating patients with nonspecific neurological symptoms. A thorough review of the patient's medications, both prescription and over-the-counter, is paramount in this type of scenario. Public education and further research into effective strategies for managing cannabis use disorder in the context of polypharmacy are essential for improving patient outcomes and preventing adverse events.
